# Polymeric Products in Erosion Control Applications: A Review

**DOI:** 10.3390/polym16172490

**Published:** 2024-08-31

**Authors:** Anna Markiewicz, Eugeniusz Koda, Marta Kiraga, Grzegorz Wrzesiński, Klementyna Kozanka, Maurycy Naliwajko, Magdalena Daria Vaverková

**Affiliations:** 1 Institute of Civil Engineering, Warsaw University of Life Sciences-SGGW, Nowoursynowska 159, 02 776 Warsaw, Poland; eugeniusz_koda@sggw.edu.pl (E.K.); marta_kiraga@sggw.edu.pl (M.K.); grzegorz_wrzesinski@sggw.edu.pl (G.W.); klementynawieczorek@gmail.com (K.K.); maurycynaliwajko@gmail.com (M.N.); 2 Department of Applied and Landscape Ecology, Faculty of AgriSciences, Mendel University in Brno, Zemědělská 1, 613 00 Brno, Czech Republic

**Keywords:** polymers, geosynthetics, geotextile filters, hydrotechnical engineering, slope, channel

## Abstract

Among the various types of polymeric materials, geosynthetics deserve special attention. A geosynthetic is a product made from synthetic polymers that is embedded in soils for various purposes. There are some basic functions of geosynthetics, namely, erosion control, filtration, drainage, separation, reinforcement, containment, barrier, and protection. Geosynthetics for erosion control are very effective in preventing or limiting soil loss by water erosion on slopes or river/channel banks. Where the current line runs through the undercut area of the slope, the curvature of the arch is increased. If this phenomenon is undesirable, the meander arch should be protected from erosion processes. The combination of geosynthetics provides the best resistance to erosion. In addition to external erosion, internal erosion of soils is also a negative phenomenon. Internal erosion refers to any process by which soil particles are eroded from within or beneath a water-retaining structure. Geosynthetics, particularly geotextiles, are used to prevent internal erosion of soils in contact with the filters. Therefore, the main objective of this review paper is to address the many ways in which geosynthetics are used for erosion control (internal and external). Many examples of hydrotechnical and civil engineering applications of geosynthetics will be presented.

## 1. Introduction

Erosion is a generic term used to describe the recession or lowering of landforms caused by the movement of natural processes, sometimes intensified by human activity [1,2,3]. Erosion is a natural process that is part of the geological cycle throughout the hydrological system, and human erosion control measures only serve to mitigate the process. Undoubtedly, erosion is a problem that can cause damage to hydrotechnical and civil engineering structures. There are many causes of slope and coastal erosion. Shoreline changes can occur in response to short-term phenomena such as wave action, winds, and storms, or in response to long-term glacial cycles and tectonic activity [4,5,6]. Human activities along the coast, such as port development and land reclamation within river basins and offshore, often intensify coastal erosion in many places [7,8]. Wind erosion occurs in the form of deflation and corrosion. Deflation is the phenomenon of the wind blowing fine particles of rock material out of large areas, and corrasion is the process of grinding, scuffing and smoothing the rock surface due to the impact of sand grains carried by the wind. Deflation removes fine soil particles, which often form a protective layer that stabilizes the slope. When these particles are removed, a less stable or more permeable layer is exposed, which can lead to an increase in the slope’s susceptibility to further erosion and landslides. The corrasion phenomenon has negative effects, as it contributes to the degradation of landscapes, the weakening of natural or man-made structures, and the alteration of soil structure, which can affect land stability and lead to landslides. Water erosion can be divided into the following subtypes: ablation (rain) erosion, fluvial erosion (including secular, local, bottom and lateral erosion) and marine erosion. Secular (sequential) erosion can be defined as erosion occurring along the entire length of the bed continuously, although with varying intensity, resulting from the water flow rate. The extent of damage caused by erosion varies and depends on several factors: kinetic energy of the water [9,10]; soil fragility [11]; structure fragility [12]; soil identification errors [13]; design and constructions errors [14,15]; and the material application [16,17,18,19].

It is interesting to note that erosion is a problem that has received increasing attention in recent times. It has led to efforts to manage coastal erosion phenomena and to restore coastal capacity. The problem of erosion is aggravated when the solutions applied are improperly designed and constructed. Sometimes, this is due to insufficient knowledge of coastal processes or reinforcement methods. Traditional strengthening methods such as nailing or anchoring are effective, but can be environmentally unfriendly and expensive [20].

Internal soil erosion is a major problem in addition to slope erosion, coastal erosion, and riverbank erosion. Today, more than 30% of dam failures are caused by this phenomenon [21]. Internal soil erosion is caused by the erosion of soil particles due to water infiltration through the structure. A flow of water through the soil leads to the formation of holes. This results in structural failure. For that reason, proper design of the structure is essential. Engineers should be aware of the properties of materials used in earthen structures [22,23].

Among the many different materials used in erosion control applications, geosynthetics deserve attention. A geosynthetic is defined as a planar product made of a polymeric material that is used with soil or other geotechnical-related material as an integral part of hydrotechnical, environmental, or civil engineering structures [24,25]. The most common polymers used in the manufacturing of geosynthetics are presented in Table 1.

In hydrotechnical structures, geosynthetics are mainly used as one of the elements in wave protection systems, anti-erosion reinforcement on slopes, embankments and excavation slopes, slopes along rivers and streams and polders, retention basins, ditches and all kinds of drains or devices that lower the water surface table and prevent the phenomenon of hydraulic failure on the downstream side of dams. There are many advantages of using geosynthetics [26,27,28]:

- Low cost for many applications;
- Quick protection against erosion problems;
- Easy installation in many structures;
- Design methodologies are obtainable;
- A wide variety of geosynthetic products are available to meet specific needs.

Geosynthetics are classified into four major groups: geotextiles, geotextile-related products, geosynthetic barriers and geocomposites [25].

**Figure 1:**
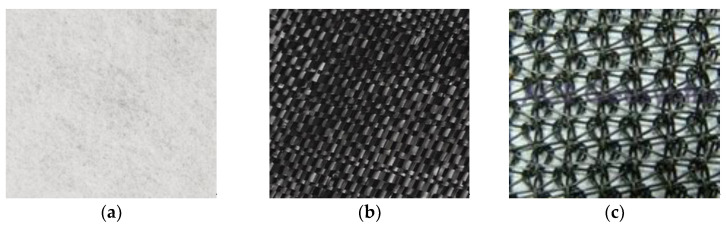
Sample of (**a**) nonwoven geotextile; (**b**) woven geotextile; (**c**) knitted geotextile.

Geotextiles are planar, permeable, polymeric textile materials used in contact with soil in civil engineering applications. The type of geotextile is determined by the method used to assemble the filaments or tapes into the planar structure. For this reason, these materials are classified as in [25] (Figure 1):

- Nonwoven geotextile—a geotextile made of randomly or directionally oriented fibers thermally and/or mechanically and/or chemically bonded;
- Woven geotextile—a geotextile produced by interlacing at right angles sets of filaments, yarns or tapes;
- Knitted geotextile—a geotextile produced by interloping one or more yarns or filaments.

**Figure 2:**
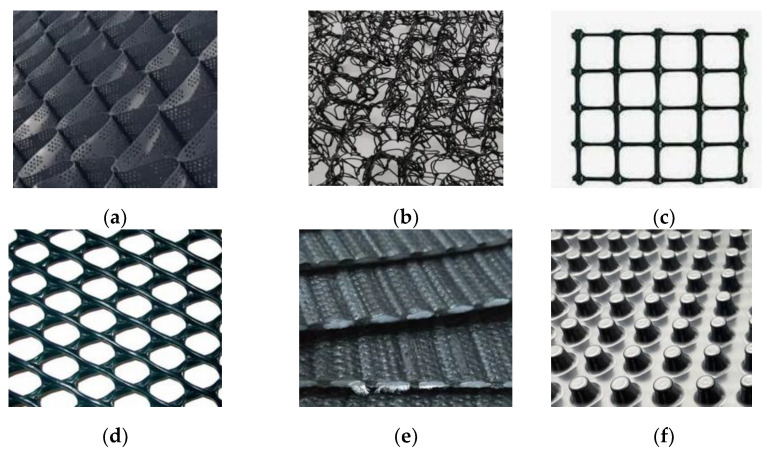
Sample of: (**a**) geocell; (**b**) geomat; (**c**) geogrid; (**d**) geonet; (**e**) geostrip; (**f**) geospacer.

Geotextile-related products are planar, permeable, polymeric materials used in contact with the ground in civil engineering applications that do not meet the definition of a geotextile. Geotextile-related products are classified as follows [25] (Figure 2):

- Geocell—a three-dimensional, permeable, polymeric honeycomb or web structure, made from strips of geomembranes linked alternating;
- Geomat—a three-dimensional, permeable structure, made of polymeric monofilaments mechanically and/or chemically and/or thermally bonded;
- Geogrid—a planar, polymeric structure consisting of a regular open network of integrally connected, tensile elements, which may be linked by bonding, extrusion or interlacing, whose openings are larger than the constituents;
- Geonet—a planar, polymeric material consisting of a regular dense network of integrally connected parallel sets of ribs overlying similar sets at various angles;
- Geostrip—a polymeric material in the form of a strip of width not more than 200 mm, used in contact with soil in civil engineering applications;
- Geospacer—a three-dimensional polymeric structure with an interconnected air space in between;
- Geoblanket—a permeable structure of loose fibers and geosynthetic elements bonded together to form a continuous sheet.

**Figure 3:**
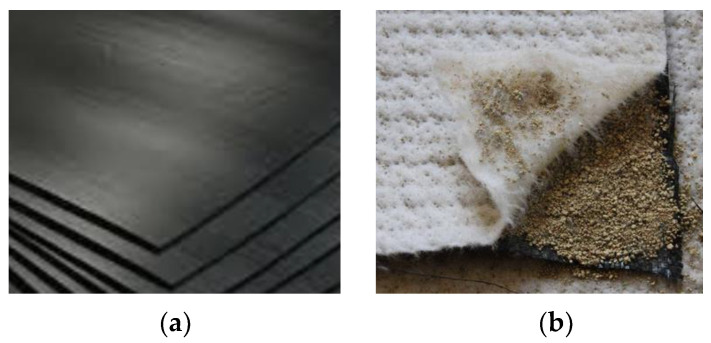
Samples of: (**a**) geomembrane; (**b**) geosynthetic clay layers.

Geosynthetic barriers prevent infiltration, protect groundwater, and guard against seepage loss. There are three barrier options: geosynthetic polymeric barriers known as geomembranes, in which the barrier function is essentially provided by polymers, geosynthetic bituminous barriers, in which the barrier function is essentially performed by bitumen, and geosynthetic clay liners, in which the barrier function is essentially fulfilled by clay [25] (Figure 3).

**Figure 4:**
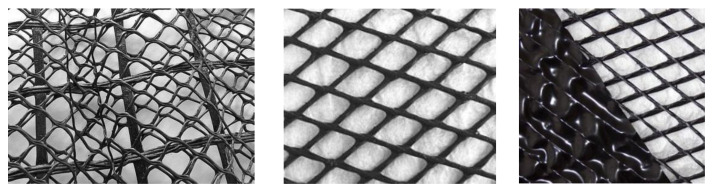
Samples of geocomposites.

Geocomposites consist of one or more geosynthetics [25], in particular a geomat, geonet, geogrid, geotextile, or geomembrane with another material (Figure 4).

The types of geosynthetics described above and the types of polymers used in their manufacture are shown in Table 2.

The primary function defines geosynthetic applications, but these materials can and most often do perform more than one function simultaneously [24,25,29,30,31,32,33,34]:

- Surface erosion control—preventing or limiting soil movement at the surface of a slope due to wind, water, rainfall, etc.;
- Filtration—allowing cross-plane flow and retaining upstream particles;
- Drainage—providing in-plane flow and retaining upstream particles;
- Separation—separating dissimilar materials, especially soils with different grain size distributions;
- Reinforcement—providing tensile strength to soils;
- Barrier—preventing the escape of liquids, gases, or solids;
- Protection—preventing local damage to a given material or material.

**Figure 5:**
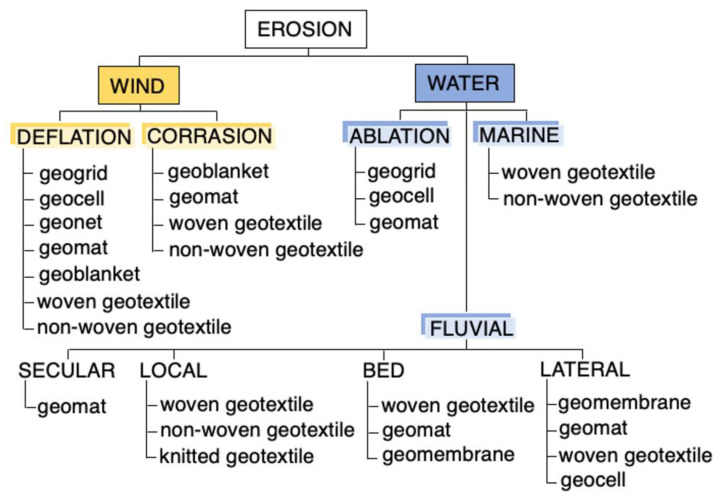
Erosion types and geosynthetic solutions (own elaboration based on [4,9,15,16,18,33,34]).

The first application of geosynthetics for erosion control began in the early 1970s. The first geotextiles were used to prevent erosion under hard armor caused by rainfall, groundwater seepage, and wave action [35]. Among many geosynthetics, geosynthetic containers, geocells and geomats are commonly used in external erosion control function. These materials are designed to reinforce small installations and extend the erosion-control limits of vegetation on slopes. Geomats are used where slopes have a steepness of 1:1.5–1:2 (Figure 5). In addition, geosystems play a key role in coastal protection, while nonwoven geotextiles are used to prevent internal erosion [31,36,37,38].

The literature, technical catalogues, and marketing efforts focus on the advantages of geosynthetic materials. However, use comes with certain limitations that can affect the sustainability and effectiveness of projects. One of the main limitations of geosynthetics is their biodegradability. Most geosynthetics are made of polymers that do not readily biodegrade. Depending on environmental conditions, such as sunlight, humidity and temperature, these materials can gradually degrade, losing their mechanical and structural properties. Some materials, such as geocells, are exposed to direct sunlight. The use of cover layers and the addition of UV stabilizers to polymers can extend the life of geosynthetics, protecting them from the harmful effects of sunlight. Damage to geosynthetics caused by UV exposure can be observed through changes in their appearance and in the performance of the material. The most noticeable changes include a partial loss of strength and a reduction in the material’s density over time.

Long-term geosynthetics in the environment can lead to the accumulation of microplastics, which enter the soil and groundwater, affecting the health of ecosystems and people. The right direction here seems to be not to exceed the duration of a given material, declared by the manufacturer, and then reuse. In addition, the importance of researching the impact of microplastics and developing methods to monitor and remove them from the environment should be emphasized.

Geosynthetics can degrade under extreme conditions, such as very low or high temperatures, high humidity, the presence of aggressive chemicals, or mechanical stresses. Such conditions can lead to cracking, loss of elasticity, or reduced tensile strength. The selection of suitable geosynthetics should be preceded by a thorough recognition of the conditions at the site of potential incorporation. Emphasized here is the performance of multi-criterion expertise, including a field inspection.

Regular quality checks at the installation stage, including material tests and technical inspections, can detect potential problems before they affect the entire structure. A high workmanship regime and the use of trained personnel are crucial here. Also, during use, materials should undergo regular maintenance and monitoring.

This article presents various applications of geosynthetics for erosion control of riverbanks, slopes, and channels. Much attention is also given to the use of geosynthetics for internal erosion control. The considerations are supported by numerous examples of engineering structures to demonstrate that modern materials such as geosynthetics perform well in erosion control.

## 2. Geosynthetics in External Erosion Control

Erosion control is a key element in maintaining rivers as waterways, especially in relation to their cross sections. The riverbed and to a lesser extent the stream banks are the areas of greatest erosion stress, while the higher areas are subject only to little erosion. The stability of river and canal banks is extremely important because of their frequent passage through densely populated areas. During periods of increased precipitation, water can flood adjacent valleys, causing property damage. To reduce the area subject to flooding and to protect property, the construction of levees is often used. These structures raise the natural banks of the river, allowing more capacity in the riverbed. However, concentrated flow between embankments can generate increased flow velocity, especially under turbulent flow conditions, further increasing erosion of banks and the riverbed. The uneven distribution of velocity both in the plane and in the cross section, as well as its pulsations, can intensify erosion processes [39]. However, geosynthetic materials have a smooth surface, which results in a lower Manning’s coefficient, allowing for greater capacity [40,41].

The drop in water level caused by the operation of weirs can have a negative effect on banks’ stability. A sharp drop in the water level causes an excessive increase in pore water pressure in the soil, which depends on the ratio of the flow velocity to the hydraulic conductivity of the soil. To reduce the negative effects, it is necessary either to reduce the effect of the flowing water or increase the resistance of the soil. The first approach can be described as “active” measures, while increasing the resistance is a “passive” approach [42].

Active methods include all activities that alter the flow pattern of water or reduce the intensity of riffles. This includes most hydroengineering structures introduced into the river flume. It should be noted, however, that these structures themselves may require passive protection, for example, by strengthening the slope at the downstream location of a weir.

The most common method of controlling stream erosion is to separate the water from the soil and prevent runoff. This can be achieved through the use of impermeable liners, which eliminate the filtration of water into the ground while protecting the subsoil from erosion, especially if it is erodible and often alluvial. In practice, various combinations of sealing layers are used, such as concrete-filled mattresses. However, special attention should be paid to the risk of mechanical damage to such materials [43,44,45].

Riprap is the most common material used as a bank and riverbed protection layer to control erosion and scour [46]. It can be used for both continuous hydraulic structures, such as thresholds or weirs, and point-like structures, such as bridge pillars. Concrete fortifications, whether prefabricated or cast in place, are less commonly used. An alternative to these “rigid” fortifications are geosynthetic containers. These are versatile elements made of geosynthetic fabric or nonwoven fabric, resembling traditional rock or concrete elements in terms of hydraulic stability. Importantly, they have a high degree of formability, allowing them to be tailored to specific project requirements. Geosynthetic containers can be filled with soil material on-site, significantly reducing construction costs [47].

To minimize the thickness of the protective layer (armor layer), but at the same time provide the best possible resistance to hydraulic action, it is recommended to use combined layers. Examples of such solutions are gabions, stone or concrete mattresses, partially grouted ballast or interlocking concrete elements. The use of planting geogrids, especially with creeping plants, can also be an effective way to further aid stabilization [48]. It should be emphasized that the best way to strengthen the slope and protect the soil from erosion is to plant it with deep-rooting plants. Rooted soil becomes more compact, and its particles are much less likely to be blown away by the wind (air erosion) and washed away by running water (water erosion). A properly planted slope is stable and does not change its shape. This ensures the safety of objects placed on the top of the slope, from which the ground does not fill in, as well as those placed under the slope, which could be buried by sliding soil. All or part of this can be achieved by biological reinforcement with the root system of plants. The soil reinforced by roots becomes more compact, and its fines are much less susceptible to air and water erosion. The riverbed and to a lesser extent the stream banks are the areas most exposed to erosion, while the higher areas are only periodically exposed to erosion.

It is important to realize that plants on slopes face difficult conditions for growth due to the slope of the ground, but also due to the lack of sufficient water in the soil. Most of the water is subject to the phenomenon of surface and subsurface runoff, which leads to the destruction of the slope and water runoff. Therefore, in many cases, they should be equipped with a special irrigation system.

Increasingly, erosion control structures must take environmental considerations into account and consider options that are closer to nature. When introducing new elements, the stabilized hydrodynamic balance of the riverbed should be considered. If well planned, construction using living materials and bioengineering measures can meet both technical and environmental requirements, perhaps in combination with engineering measures, such as geotextile reinforced fascine banks used to protect against erosion and scour in large rivers. Typically, a geotextile is used as the base. A composite geotextile is often used: fascine mattresses provide filtration and stabilization through laterally attached strips.

Optimal levels of biological and biotechnical protection can only be achieved when plants are able to successfully take root and grow without stress. Therefore, most systems initially require additional protection. Shelter can be provided either by reducing the hydraulic impact on the bank or by installing additional elements to protect the living material. Suitable structures may include riprap placed in the embankment parallel to the shore, a woven fence, or a row of wooden stakes.

**Figure 6:**
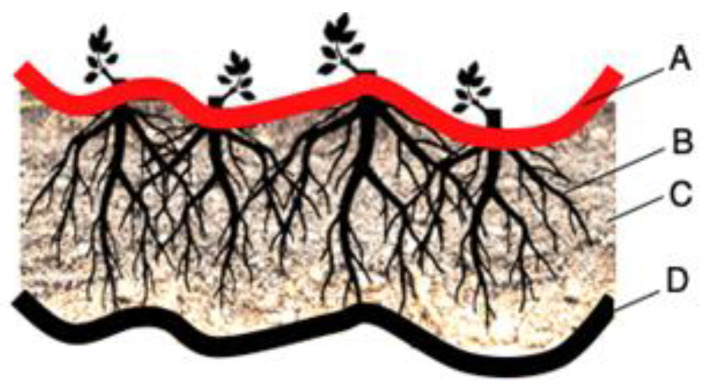
Willow root growth through geotextile sheet, where: A—woven geotextile, B—willow root; C—soil body; D—filter layer.

If the roots of the protective plants reach far enough, they may be able to provide the filtering function needed to stop erosion. However, in the beginning, during the vegetation development stage, an additional filter is needed. Ideally, this should be a geotextile filter, as granular filters (which require a certain thickness) will not provide sufficient plant nutrition (Figure 6). An alternative protection system can be installed with pregrown vegetation in gabions or mats. Such elements are heavy enough to resist hydraulic action and provide the necessary soil loading.

Other anti-erosion solutions that combine geosynthetics and natural materials are sodding, turfing, riprap, stone riprap, gabion fortifications and fascine bundle fortifications. Sodding is mainly used in the upper part of the slope (above the average annual water level) on convex curves and straight sections. To obtain a lawn with an optimally developed root system, it is necessary to select a grass mixture that is appropriate for the soil and water conditions. The mixture should contain 60% low grasses and 40% tall grasses [49]. It is advisable to use the resources of existing vegetation as mother plants and use them for new plantings. This ensures the introduction into the flume of the watercourse of species appropriate to local climatic and soil conditions, as well as the landscape, and usually leads to the preservation of species diversity. Important for the development of riverbank vegetation is the protection of existing vegetation, mainly through appropriate maintenance, replenishment, and sparing it during regulatory and maintenance work. If local bank conditions are unfavorable for the natural development of vegetation, for example, in the case of frequent floods, geosynthetic mats can be used, giving plants a chance to take root in difficult conditions. The erosion control sheet may need to be fixed to the ground, usually with pegs. Riparian vegetation can be used where its expansion will not cause unacceptable deterioration of flow conditions—on watercourses with a water table width ≥ 2 m and a depth of 0.5 m.

Turf is another treatment that can provide a high degree of erosion control. The resistance of vegetation to erosion depends mainly on the turf, which must be dense enough to keep soil particles out and must be strong enough to keep bare spots out of the vegetated area. To increase the strength, stone reinforcements or a layer of asphalt are often used, covered with soil on which grass can grow to restore the “green” appearance of the embankment. The most common methods of sodding are: sheepskin—made over the entire slope, on the wall—made from the level of the longest-lasting water to the top edge, in places where there are local potholes and groundwater seepage, and in a grid, made above the longest-lasting water if the length of the slope in cross section exceeds 3.0 m [49]. Biotextiles made of organic fibers (cotton, flax, coir) or organic fibers combined with polypropylene fibers are also used to reinforce such slopes, especially if the slope requires only periodic protection. Synthetic fine-mesh netting is often used to protect lightweight mulch material from displacement and wind erosion.

Riprap is used for slope reinforcement and erosion control of the middle and upper slopes of a riverbed with a bottom width of more than 5 m. Riprap consists of a layer of woven mulch (capable of regrowing) and wire. This type of reinforcement is always used together with bottom strip (base of the slope) reinforcement. Two types of banks are used [49]:

- Covered—wicker is laid in strips, parallel to the course of the river, nailing it with wire;
- Flat—the wicker is laid at once over the entire slope perpendicular or at a 45° angle to the direction of water flow, and then nailed with wire.

**Figure 7:**
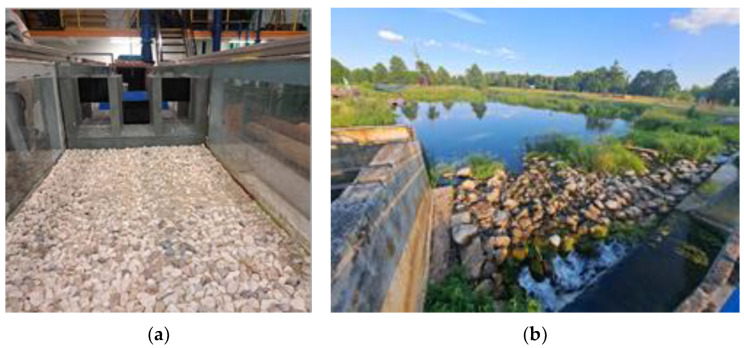
Stone riprap as reinforcement of the lower damming weir site: (**a**) laboratory model; (**b**) tailwater weir site on the Wkra River (Strzegowo, Central Poland).

Stone riprap is used for strengthening and erosion control of the lower and middle strip of the slope or local slope protection in areas particularly prone to erosion. The surcharge can be made on a gravel bed 10–15 cm thick [49,50]. Supplementing such reinforcement with wicker cuttings, “living stone riprap” is obtained. If exudates occur, the slope is additionally protected with a nonwoven fabric (laid under the bedding layer) acting as a reverse filter. Stone riprap can be a form of flexible fortification downstream of hydraulic structures (Figure 7), and its roughness can serve to dissipate energy. Stone fortifications are an exceptionally good environmental solution. They are characterized by intense siltation capability and create a habitat for many microorganisms, but also for all aquatic flora and fauna. In addition, stones in silted form are an ecotone, that is, a transitional form between the aquatic and terrestrial ecosystems.

**Figure 8:**
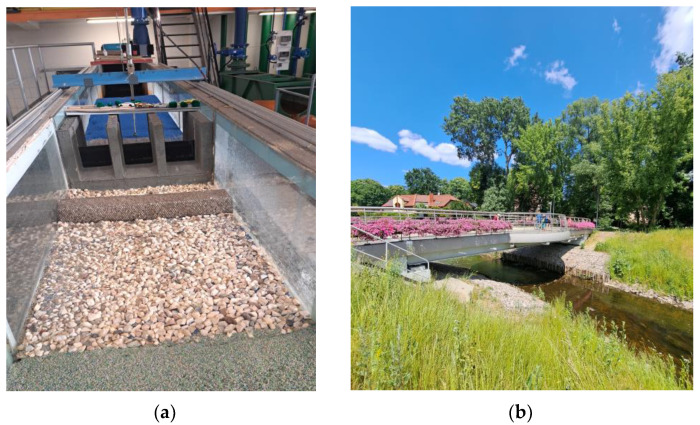
Gabions in hydrotechnical structures: (**a**) laboratory model of gabion threshold; (**b**) bank reinforcement in the area of the bridge.

Gabion fortifications are recommended especially in the channels of watercourses where there are high water velocities or undulations, and on slopes made of soils prone to seepage. Securing high, steep slopes with mesh and stone elements gives good results. Mesh-stone structures (Figure 8) of various sizes and shapes made of reinforced stone riprap are used in hydraulic engineering because of their high strength, resistance to external factors and natural properties [49,51]. These structures are flexible and susceptible to deformation, so they adapt to the shape of the substrate and achieve better stability, without changes in strength. Gabions are most commonly used to reinforce bank and stream beds at high flow velocities, in areas where soil or bank erosion may occur. Due to water erosion, it is crucial to place gabions on inverted filters. They can perform a variety of hydraulic engineering tasks, the most popular of which are: protecting the slopes of embankments from landslides, strengthening the banks of rivers, canals, and reservoirs, and filling in the gaps, holes and cavities that form in river channels and fortification structures. Bank bands, bridge pillar covers and sills are made of gabions. Hydrotechnical structures made of gabions have a positive effect on the oxygen balance of the river, causing strong aeration of the water [51].

Fascine bundle fortifications can be used on straight sections and concave curves of smaller rivers, but only where no deep erosion is observed. The upper edges of such fortifications should end at the level of normal or mean low water. Bundle fortification of the base of the slope can be made of one, two, three, and in local depressions, even five fascine bundles. The rhizomes of rushes (e.g., reed, calamus) can be inserted between the fascine branches. Bundles with the addition of nonwoven filter fabric are used when there is non-cohesive loose soil at the bottom of the watercourse or when it is necessary to collect seeping waters at the foot of the slope. Wire mesh or natural fiber rolls can be used to reinforce the base of the slope.

**Figure 9:**
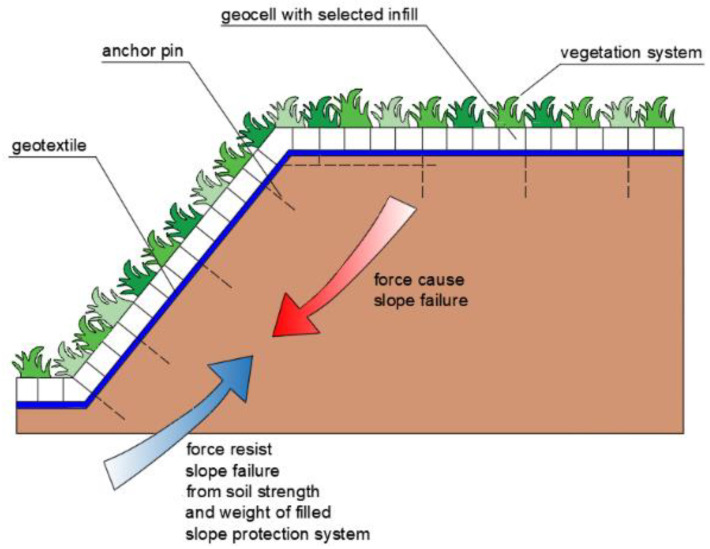
Geocell slope stabilization.

In addition to the above, geocells are also commonly used in the external anti-erosion function. Geocells above the slope provide a stable environment for the growth of vegetation. It improves the stability of the slope. However, for the slope at the initial stage of construction, the system of vegetation is not strong enough and consequently is more vulnerable to water or rain damage. For this reason, geocells should be used to build the topsoil to reduce the loss of slope soil. Geocell systems utilize the effect of geosynthetics on the topsoil and combine the vegetation root system on the slope surface to form the entire lateral bond (Figure 9). In addition, gravel, a combination of gravel and soil or even concrete can be used as a backfill material [52,53].

In recent years, many theoretical analyses and practical research have been conducted on the application of geocells in erosion control [54,55,56,57]. Based on the comparison of the anti-erosion effect between many slope protection methods, Zhang et al. (2012) proposed that the soil consolidation effect of the geosynthetic is better than that of the three-dimensional mesh mat and wire mesh [58]. Also, Yan et al. [59] confirmed that geocells have an anti-erosion protection effect on the residual soil slope. Moreover, based on the drainage project in the flight area of Beijing New Airport, Zhao and Yin [53] have observed that the pore water pressure of the slope soil is reduced after the installation of geocells. Song et al. [60] have found that geocell-reinforced slopes reduced soil erosion by as much as 70%. The only thing that might be questionable is the fact that most of the geocell material used in civil and hydrotechnical engineering is high-density polyethylene, which is difficult to decompose under natural conditions. However, according to the above researchers, geocells are widely used in slope protection projects. They can also be used with grass planting and three-dimensional mesh mats to achieve slope protection effect.

**Figure 10:**
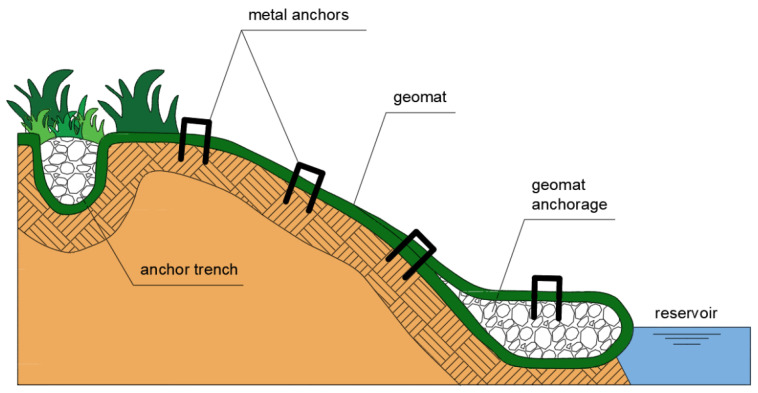
Geomat slope stabilization (adapted from [63]).

Geosynthetic erosion control mats also provide erosion control before vegetation growth. Finally, geomats become interwoven with the vegetation, enhancing the performance of the vegetation (Figure 10). Due to its open surface, the geomat can be filled with soil over its entire area and depth, which results in rapid vegetation of the slopes and guarantees erosion control. It can be concluded that the anti-erosion mechanism and the effects of its use are similar in analyzed cases [61,62,63]. Tan et al. [64] studied the shear behavior of soils reinforced with geomats. The obtained results showed that the addition of a geomat to the soil improved its shear strength. Melo et al. [65] conducted studies with four different types of geomats used in rain simulation tests. The masses per unit area and the tensile strengths of the tested samples were equal to 400, 400, 400, 520 g/m^2^ and 0.69, 0.70, 0.38, 3.00 kN/m, respectively. The research showed that all geomats tested reduced the amount of soil eroded.

**Figure 11:**
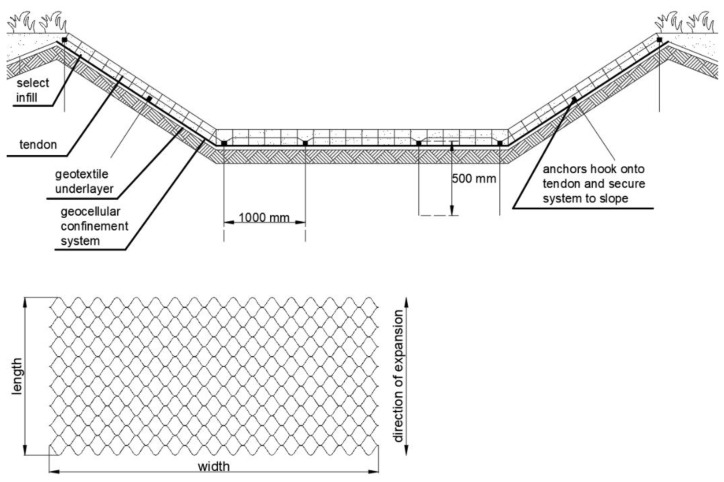
Example of installation of geocells in channel bank erosion control.

Geocell and geomat systems are also suitable for the passive protection of river and channel banks (Figure 11). Erosion on channel banks is caused by the shear stresses exerted by the flow. Uncontrolled erosion can cause levee failure, resulting in flooding. Water flow in rivers produces shear stresses that are proportional to water depth and velocity. This can remove soil particles and excavate deeper into the channel bottom and sides [66,67].

In the design process, it should be taken into account that a geocell/geomat–soil system must resist sliding forces from the infill soil and the slope dimension imposed upon it [68]. However, calculation for the design of bank protection can be performed using two methods—considering the acceptable speed of water and the allowable shear stresses [67]:

V < V_all_/FS, (1)

T < T_all_/FS, (2)

where V_all_ and T_all_ are the water velocity at which the soil particles begin the movements and the shear stress produced by the current, respectively. FS is the factor of safety.

**Figure 12:**
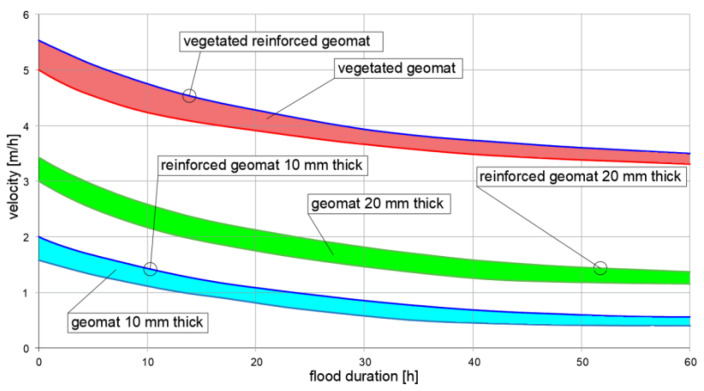
The water velocity at which the soil particles begin the movements graphs (adapted from [67]).

**Figure 13:**
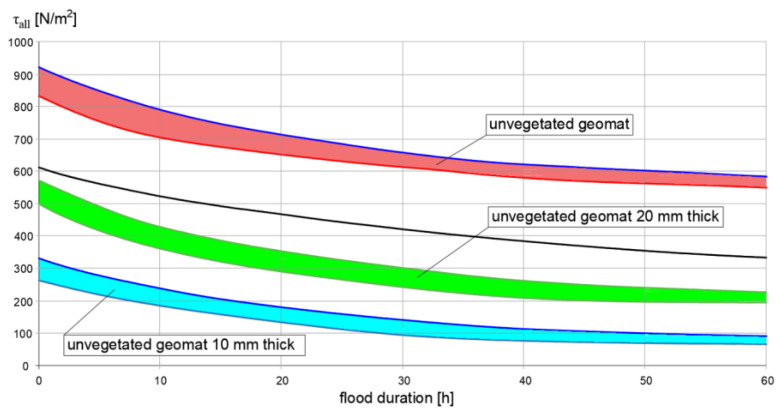
The shear strength graphs (adapted from [67]).

In general, the stability check should be performed at the very end of the work and when the vegetation is fully grown. Figure 12 shows a V_all_ graph. The value of the parameter is a function of the duration of the flood event flow for a family of geomats. The lower zones refer to non-vegetated geomats and the upper zone refers to vegetated geomats. The upper limit shows V_all_ for fortified geomats. These graphs can be provided by manufacturers for each type of product. Similar graphs can be prepared for T_all_ (Figure 13) [67].

Moreover, the interaction between soils and geosynthetics is of paramount importance when these materials are used as reinforcement. In engineering applications of cellular geosynthetic-reinforced soil, it is usually necessary to analyze the deformation or failure of the geosynthetic–soil composites. The soil–geosynthetic interaction mechanism is very complex and depends on the properties of the geosynthetic and the soil. Failure and ultimate strength increase significantly with the inclusion of geosynthetics. As reinforcement layers increase, peak and ultimate strengths increase significantly with a corresponding increase in axial strain. Ramp tests are relevant to the study of slope erosion control. Numerical analysis also allows to study the evolution of the force mobilization in a geosynthetic layer and the interface shear strength as the ramp slope increases.

### 2.1. Concept of Eroded River Sections Restoration Using Geosynthetic Materials

Regulatory measures involving the construction of eroded sections of a channel require a thorough analysis of the hydrological and geomorphological parameters of the watercourse and the genesis of the damage. This is necessary to select and use appropriate material that will adequately protect the damaged riverbed from further erosion, will not be damaged during high water levels, and will not cause possible major damage to the watercourse and the aquatic ecosystem.

One of the most important pieces of information needed to choose the right material for the stream restoration is what kind of native soil is present in the stream and the eroded section, but also in the valley area. The choice of the method and material of reinforcement is influenced here by whether the soils lying in the bed and slopes are cohesive, permeable soils, what value their filtration coefficient reaches, and what degree or rate of compaction they achieve. As for the geological structure of the valley, it is necessary to analyze the porosity and filtration coefficient of the soils forming it.

Further analysis of the factors determining the choice of materials for the reinforcement and construction of eroded channels should include the question of the degree and source of pollution of the watercourse, which should be cited. In the simplest terms, surface water pollution can be divided into two groups—natural pollution and artificial pollution. The occurrence of pollution from the former group is usually caused by natural processes in the environment. Examples include atmospheric precipitation (acid rain), soil-forming, or geological processes. They lead to changes in the water chemistry, for example, by changing the pH of water, the iron content, but also by increasing the amount of mineral debris. The second group of pollutants—anthropogenic pollutants—are characterized by anthropogenic origin. The change in water chemistry is most often caused by fertilizers, pesticides, manure or slurry, which enter the watercourse along with surface runoff. These factors are mainly due to human agricultural activities, but also to industrial intensification. Polluted water can cause erosion not only in the natural parts of the channel but also (especially) in the built-up parts. For this reason, the material selected for the development of an eroded section should be durable and resistant to changes in water chemistry.

#### 2.1.1. Case A—Fuzzy Shoreline, Advanced Lateral Erosion

**Figure 14:**
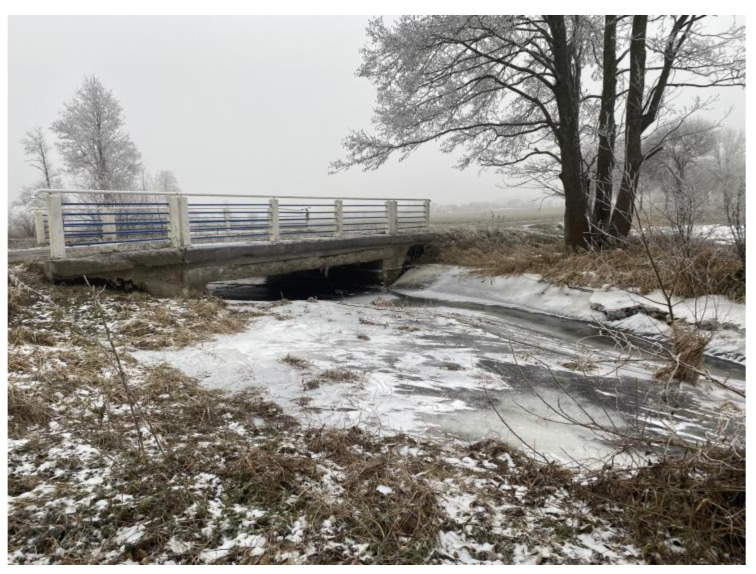
Case A—fuzzy shoreline, advanced lateral erosion.

The damaged section of the river is located in a small bend. The destruction due to a lateral erosion occurred on the right concave bank as a result of the landslide of earth masses from the entire height of the bank, forming an escarpment with a steep slope of about 1:0.25 and a length of about 25 m was formed (Figure 14). However, no significant deep erosion or accumulation of landslide earth material in the soil was observed. The left bank of the watercourse, convex, was preserved in an undamaged condition, and the slope of the bank was about 1:1.5.

**Figure 15:**
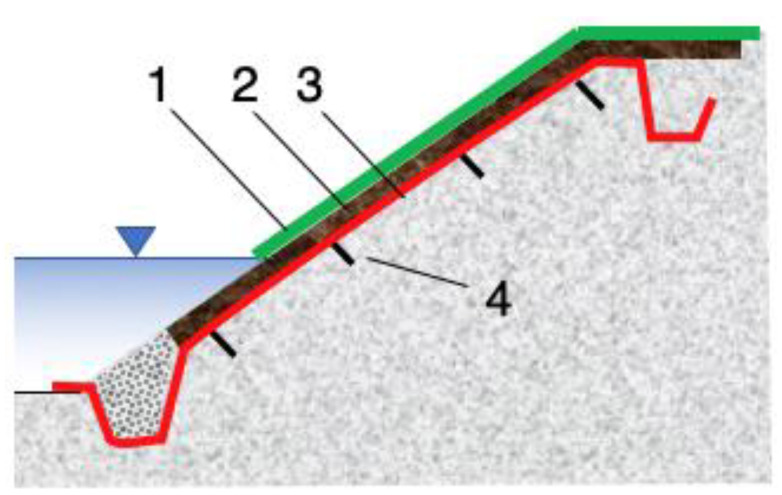
Diagram of the proposed solution for the reconstruction of the damaged bank A with the use of synthetic materials, where 1—seeding with grass mixture; 2—biaxial rectangular geogrid with tensile strength 30 kN/m and thickness 2 mm filled with soil; 3—high tenacity polyester geotextile fabric, with tensile strength 30 kN/m and 1 mm thickness; 4—fixing pins.

In this case, it is proposed to reconstruct the slope by constructing an embankment of soil compacted in layers with a maximum thickness of 30 cm, as in the proposal with natural materials. The reinforcement of the slope and its base was proposed by using geosynthetic materials—geotextile and cellular geogrid. The geotextile will have a protective and reinforcing function. It should cover the entire height of the slope, and its fixing should be accomplished by driving fixing pins and digging the upper part of the geosynthetic into the crown of the scarp and the lower part into the bottom of the watercourse to a depth of about 60 cm (Figure 15). On top of the geotextile filter fabric, it is proposed to install another geosynthetic in the form of a cellular geogrid. As with the geotextile, this material should be secured with anchors (fixing pins).

#### 2.1.2. Case B—Scarp Trampled by Animals

The riverbed, which has been eroded by cattle, requires maintenance measures in the form of rebuilding the trampled slope and protecting it from further degradation by animals. While the current state of the riverbed is not threatening in itself and there is no need to rebuild it, it is necessary to protect the river from contact with cattle. This is since cows entering the riverbed directly pollute it with their feces. This causes significant local (downstream) deterioration of the ecological and chemical state of the watercourse. This is due to the high concentration of nitrogen and phosphorus in the water, which leads to the intensification of eutrophication processes. Based on the results of the bottom soundings, it was found that the right bank of the watercourse, on the side where the land is used as pasture, is intensively softened and the channel is shallow.

For the geosynthetic materials for the analyzed damage, it is proposed to use the same materials as in the previously analyzed case with one important difference. The technology of the work and the materials used will differ in the final stage of implementation. Reconstruction of the channel should begin with the laying and proper compaction of the soil layers. Then, the formed embankment should be covered with a layer of geotextile fabric restrained in the crown of the slope and the bottom of the watercourse by digging in, and additionally secured against sliding by three horizontal rows of anchor pins at 0.7 m intervals, and vertical ones at 1 m intervals. The whole procedure should be carried out analogously to the previous case.

The next step is to lay the cellular geogrid and secure it against sliding with pins installed in the upper parts of the cells in every third cell in the vertical direction and in every second cell in the horizontal direction, as well as in every cell at the ends of the structure. The last step is to fill the cells with geosynthetics. This solution proposes the use of pebble material for this purpose. This will minimize the proliferation of riparian vegetation, which is food for cattle and makes it more difficult for them to access the channel.

#### 2.1.3. Case C—Degradation by Beavers

**Figure 16:**
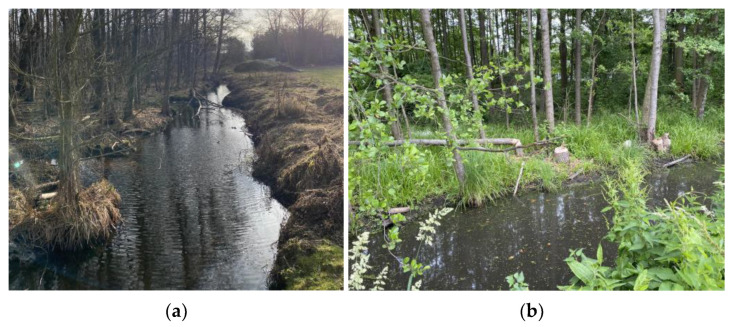
The watercourse bed and valley in damaged area C: (**a**) under conditions of poor winter vegetation; (**b**) observation during summer field inspection.

For many years, the study area was destroyed by beaver activity. The displacement of the beavers caused the problem to subside in the form of continuous dam reconstruction and the formation of high dams. However, beaver activity has degraded the riverbed for about 100 m and the valley (Figure 16). The riverbed is heavily excavated by beavers and there are numerous burrows and cavities. The area also provides a habitat for wild boars, which also contribute to the erosion of the riverbed and valley. These factors weaken the stability of the slopes of the watercourses, which need to be secured and reinforced. During the site visit, it was observed that the riverbed is very symmetrical in shape, and the slopes are quite gentle.

The concept of reconstructing the riverbed with geosynthetic materials is also based on the initial scouring and subsequent filling of the corridors created by the beavers. However, the choice of materials to protect the riverbed from the activities of the tormenting animals is much wider here. Among the geosynthetic materials, one can choose cellular geogrids, geogrids, galvanized mesh, or geomembranes. A solution has been proposed using the third of these—galvanized mesh in combination with geotextile. Geomembranes, often used in flood control embankments, provide good protection against this type of damage. However, this material has a very low degree of permeability, which is not a good solution for a natural watercourse.

**Figure 17:**
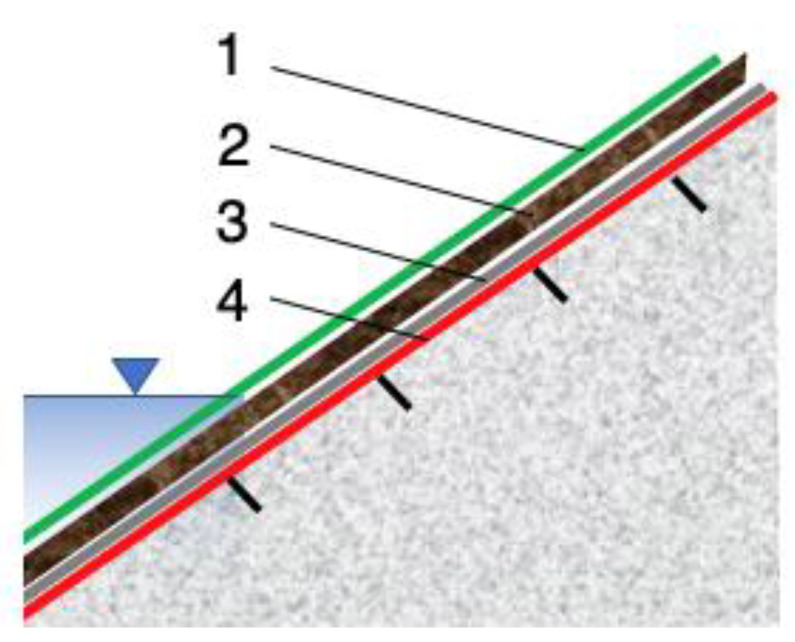
Diagram of the proposed solution for the reconstruction of the damaged bank at site C using geosynthetic materials, where 1—sowing with grass mixture; 2—humus; 3—galvanized mesh fixed with pins; 4—polypropylene (PP) spunbonded nonwoven geotextile fixed with pins with tensile strength 23 N/5 cm and 0.5 mm thickness.

The proposed solution (Figure 17) is to lay a reinforcement layer in the form of a geotextile over the entire height of the slope, with galvanized mesh on the surface. The geotextile should be attached to the slope with 30 cm long pins at intervals of 0.6 m vertically, and 1.0 m horizontally. The mesh should be selected so that the mesh size prevents animals from accessing the deeper layers of the slope and the wire diameter provides sufficient strength. Therefore, it is suggested to use a net with a mesh size of 8 cm by 10 cm and a wire diameter of 2.4 mm.

The mesh, as well as the geotextile, should be fixed with pins 30 cm long, which are used in every second eye at the crown of the slope and the bottom, and on the surface of the slope at a spacing analogous to the pins fixing the geotextile. The protection thus prepared should be covered with a layer of humus and sown with a grass mixture.

## 3. Geosynthetics in Internal Erosion Control

Internal erosion is undoubtedly a fundamental phenomenon that contributes to the failure of many drainage systems that are not visible from the outside. The phenomenon of internal erosion occurs when soil particles are eroded from the inside or underneath a water-retaining structure. To prevent internal erosion nonwoven geotextiles are commonly used. When discussing the use of geotextiles, it is also important to consider their impact on the environment, especially during their production and disposal, as well as the potential long-term impact on ecosystems. Geotextiles are most often made from recycled plastics such as polyethylene (PE) or polypropylene (PP). Their production and disposal require significant energy consumption, which can have a negative impact on air quality. The use of chemicals during production can also cause water pollution. Therefore, it is important to use modern systems during the production and disposal of geotextiles, which will minimize or eliminate factors that may negatively affect the environment. Geotextiles are not biodegradable, but are recyclable. When disposed of properly, they do not pose a significant risk to human health or the environment. The potential long-term impact of geotextiles on ecosystems is not clear. On the one hand, they are harmful to the environment, but on the other hand, they can be used sustainably. There is no doubt that geotextiles have an impact on the environment, but their role in sustainable development is often overlooked. Geotextiles can be used to improve water quality, control erosion, and reduce greenhouse gas emissions. When used correctly, they can help achieve a more sustainable future.

### 3.1. Internal Erosion in Embankment Dams

The criteria for the onset and development of internal erosion depend on the soil structure under consideration and are therefore divers. Cases of internal erosion occur mainly in the embankment, but we can also see it in the drainage systems of buildings and subsoil. There are many ways to prevent internal erosion, but due to the specificity of the article, only protection with geosynthetics, mainly geotextiles, will be discussed. Internal erosion in embankments is one of the most dangerous phenomena that can contribute to disasters. The main causes of failure are known to be overtopping (35.9%) and piping (30.5%) [69]. Damage to the embankment dams due to internal erosion is largely dependent on the physical properties of the soil materials from which the embankment was built [70,71]. Studies have shown that the rate of internal erosion depends on the following factors [72]:

- Plasticity;
- Fine soil and clay content;
- Dispersity (detachment and spreading of soil particles);
- Dry density;
- Water content;
- Mineralogy;
- Degree of saturation;
- Content of cementing materials such as oxides iron.

Furthermore, coarse-grained and non-cohesive soils erode faster at lower critical shear stresses than fine-grained soils. An increase in factors such as the plastic index, clay content, dry density, and optimum moisture content will result in lower soil erodibility, while an increase in saturation, cementing materials such as iron oxides, will result in higher soil erodibility.

To prevent this phenomenon, drainage systems with reverse filters are used. In practice, it is impossible to imagine a dam with poor drainage operating safely. The design of drainage systems for dams or landfill sites is important as it has an impact on their stability [73]. Like all technical equipment, drains and filters are subject to degradation as a result of mechanical damage, both external and internal, caused by displacements of the dam body, suffusion of soil into filters and drains, precipitation of chemical compounds (mainly manganese and iron) from the filtering water, leading to clogging, pipelines and filters, or even aging of the materials used. In general, the period of reliable operation is estimated at a maximum of 15 years.

In many facilities, especially those built for agricultural purposes, drainage systems were not installed during construction or were installed in a limited and inadequate manner. Therefore, it is often necessary to carry out renovations, i.e., renovation or reconstruction of building drainage systems in operating facilities.

Renovation works on hydrotechnical facilities are always complicated and must be carried out with particular precision in order to prevent damage to the structure, which may even lead to a construction disaster.

Most often, they have to be carried out in conditions of unfavorable filtration processes caused by drainage failure and while maintaining a normal level of damming. Unfavorable filtration processes that may occur during the renovation of hydrotechnical structures include: high pressures, hydraulic breakthroughs, suffusion, and loosening of soil. Rehabilitation of earth dams requires significant excavation of the dam body from the upstream side. This can contribute to loss of stability. In general, the process of internal erosion in dams can be divided into four phases [74]:

- Initiation of erosion;
- Continuation of erosion;
- Progression to formation and maintenance of a pipe and/or increase in seepage and pore pressures in the downstream part of the embankment or its foundation;
- Development of a breach resulting in uncontrolled release of the water from the reservoir.

There is always a reason for the initiation of erosion. For this purpose, loading conditions and location of initiation of internal erosion must be considered. The loading conditions to be considered are: frequent water level, infrequent flood, safety flood, and seismic loads. It is preferable to consider the full range of flood levels and seismic loads rather than just the extreme values. The loading condition has a great influence on the location of the initiating event and its probability of occurrence. The most probable locations according to the loading condition can be considered: upper portion of the embankment, lower portion of the embankment, along the conduit, adjacent wall, and foundation. The initiation of internal erosion can occur as a result of: backward erosion, concentrated leakage, suffusion, contact erosion and critical gradients or velocity. The continuation of the phase depends on filters capable of stopping the erosion process. The phase progression concerns the methods available for assessing progression of internal erosion. The last of the mentioned phases leads to failures and construction disasters such as: gross enlargement, unraveling or slope instability, loss of free board by crest settlement, sinkholes, and loss of the free board.

**Figure 18:**
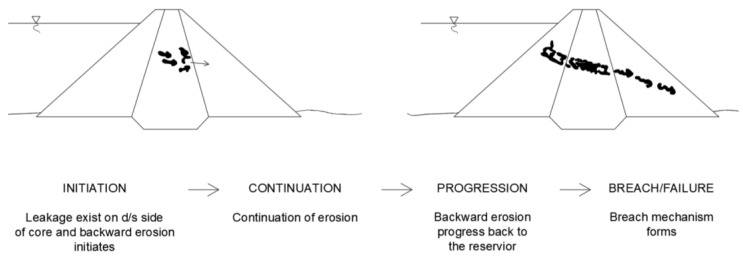
Diagram of internal erosion in the embankment by backward erosion (adapted from [75]).

**Figure 19:**
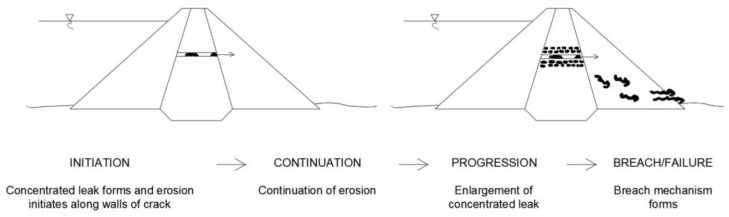
Diagram of internal erosion in the embankment in a concentrated leak (adapted from [75]).

Diagrams of individual phases of the most common causes, which are internal erosion in the embankment by backward erosion and concentrated leakage, are shown in Figure 18 and Figure 19 [75].

To determine the criteria for the initiation and development of internal erosion, laboratory tests are most often conducted to identify specific mechanisms for initiating particle migration. Most of the laboratory research conducted leads to the development of new methods for predicting the internal instability of the soil, and thus the causes of internal erosion [76,77,78,79,80,81,82,83]. Studies available in the literature indicate that when the clay content is low, erosion of sand particles occurs and, as a result, the skeleton of the sample is broken down [84].

Internal erosion is a complicated phenomenon and there is no clear way to protect embankment dams against this phenomenon. Common methods of protection include strengthening the subsoil by soil stabilization and creating anti-filtration barriers. This is one of the most common methods, next to surface and deep drainage. Each method is more or less effective. Research continues to improve the results. From the research that has been conducted, it appears that the main research is focusing on strengthening the subsoil and testing the soil in the embankment dam and around the drainage.

Soil stabilization in embankment dams is performed by adding reinforcing materials to the soil to increase the strength and properties of the soil. The most common studies in the literature involve the use of typical physical stabilizers such as: cement, lime, and fly ash [85,86,87,88,89,90,91,92,93,94,95,96,97,98,99,100,101,102].

Research on the use of chemical stabilizers has been conducted by [103,104], among others. They studied the chemical stabilization of the clayey soils against internal erosion. There are also studies available in the literature using non-standard materials such as wheat husks as stabilizers [105].

Research on soil stabilization showed that with optimal soil stabilization, a significant reduction in the erosion rate was observed, as well as an improvement in the erosion rate index and the critical erosion stress. The results also showed that the curing time increased the erosion rate index and reduced internal erosion in embankment dams. From the literature review, soil stabilization has been thoroughly investigated, and each of the above materials has demonstrated excellent stabilization properties.

Numerical approaches, including the finite element method, are also used to evaluate seepage and thus monitor the phenomenon of initiation of internal erosion in embankment dams. Existing numerical models use a generalized form of the Richards equation, in which the hydraulic head of the water is determined in terms of hydraulic conductivity [106]. The limitation of this method is that it was originally used for 2D analysis, which simplifies the analyses performed. Similar ways of modeling this phenomenon have been proposed by Alekseevich and Sergeevich [107] and Aniskin and Antonov [108]. Their methods use an analogy between Darcy flow, i.e., hydraulic head, and the differential heat conduction equation to predict the propagation and the flux of the flow through the soil. However, these methods consider steady-state conditions without considering the changes in the boundary conditions, i.e., dam water levels are considered.

Another numerical model has been proposed, among others, by Wise et al. [109], who developed the framework for using a transient thermal finite element analysis model as an analog to predict transient seepage. This model is compared to a transient analytical model to verify the approach. A sensitivity analysis showed that the time aspect of the seepage flow depends only on the medium (i.e., soil) and not on the fluid properties (permeability, porosity, and length) while the flow rate depends on the soil permeability, length, fluid viscosity, and pressure differential. It should be noted that most of the currently available numerical models are computationally expensive or assume steady-state conditions. It is expected that further development of numerical methods will take place, which will consider additional factors and thus more accurately describe the phenomenon of seepage through embankment dams, leading to internal erosion.

When analyzing internal erosion in embankment dams, it is also important to mention the wireless underground transmission sensor developed by Liang et al. [110]. This device monitors the seepage water level inside the embankment dam, which is the cause of internal erosion. The study conducted proved that the wireless underground transmission sensor was better than the water level gauge in monitoring the actual seepage phenomenon, which could play a crucial role in future early warning systems.

To date, there are relatively few examples and little research on the use of geotextiles to prevent internal erosion. They are used relatively rarely and are one of the most durable and best methods to protect against this phenomenon. The geotextiles, if properly selected and placed, will properly prevent internal erosion. The type of geotextile should be selected depending on the conditions, location, and design requirements. The geotextile must have appropriate hydraulic properties. The main parameters of geotextile materials used in internal erosion control and methods for their determination are presented in Table 3. Needle-punched nonwoven geotextile is the best choice, as it guarantees reliable and long-lasting operation. Needle-punched geotextile technology involves piercing layers of polypropylene fibers with microscopic needles equipped with hooks. As a result of this process, pores are created in the materials, allowing water and air to flow freely, which is impossible in the case of pressed materials. Due to their needle-like structure, geotextiles act as a filter, preventing small soil particles from migrating and the drain from clogging. Water then percolates through a very large number of micropores in the geotextile, which results in a larger amount of water transported compared to a perforated pipe. It should be noted that under the influence of mechanical, chemical, and biological clogging processes, the hydraulic properties of geotextiles may change, including a significant reduction in the lateral water permeability coefficient. In embankment dams, the filters protecting the drainage are most exposed to mechanical and chemical clogging processes [111,112]. For this reason, knowledge of the interaction between the soil and the geotextile is particularly important for the design of geosynthetic drainage systems. The most commonly used parameter to assess soil–geotextile compatibility is the gradient ratio. The gradient ratio is expressed as the soil–geotextile hydraulic gradient divided by the soil hydraulic gradient. The gradient ratio should not be greater than 3.0. Many laboratory gradient ratio test results for different types of nonwoven geotextiles have been presented by Markiewicz et al. [28].

Research on changes in the filtration properties of geotextiles used in drainage systems of embankment dams was carried out, among others, by Miszkowska and Koda [34]. This research presents the results of analyses obtained from the laboratory tests of water permeability characteristics normal to the plane of nonwoven geotextiles after 22 years of exploitation in the embankment dam located in Białobrzegi (Poland). Also, the parameters of unworn and after 7 years of exploitation obtained earlier were compared with the research results. Based on the conducted research, it was also found that despite taking samples for testing from 1 m^2^ of a sheet of used geotextile, the clogging process does not run parallel and uniformly over the entire surface and volume of the material. At the same time, attention was drawn to the fact that in the case of geotextiles that are several decades old, they may be heterogeneous due to the production technologies used several decades ago. Therefore, tests should be conducted on samples taken from different places.

**Figure 20:**
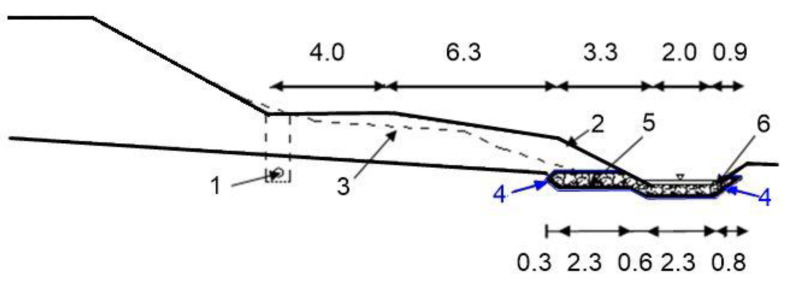
Protection of the dam ditch and stone drainage with geotextile at the Białobrzegi dam [28]: 1—old drainage; 2—ground level after renovation; 3—ground level before renovation; 4—nonwoven geotextile; 5—stone drainage (thickness 0.3 m); 6—crushed stone (thickness 0.2 m).

An example of the reconstruction of drainage systems using geosynthetics in exploited embankment dams is the dam in Białobrzegi [113]. This dam was built in 1959–1962. The length of the dam is 1.57 km, the width of the crown is 3 m, the inclination of the upstream slope is 1:1.5, the inclination of the downstream slope is 1:3. The dam was founded in unfavorable subsoil conditions, because under the surface of the sands, which were approximately 5 m thick, there were gravels with a direct connection to the reservoir. This arrangement caused a hydraulic breakthrough in the sand layer and the phenomenon of contact suffusion, which involved carrying soil from the bottom of the sands through the breakthrough crater at the bottom of the ditch. As a result of contact suffusion, damage occurred that constituted a threat to the dam. The dam renovation was carried out while maintaining full damming in the reservoir, which constituted a significant difficulty and threat. During the renovation of the dam, it was assumed that the existing drainage, due to its wear and tear, would be treated as an additional element. All the water filtering through the dam would be taken over by a rebuilt ditch and additional drainage. The ditch was protected with a 0.2 m-thick layer of riprap. Since the works were carried out underwater on unstable soil, the reverse filter was made of 8 mm-thick geotextile. In this case, a PP andPET geotextile with the following parameters was used: mass per unit area 460 g/m^2^; thickness 4.58 mm; tensile force in the machine direction (MD) 500 N; tensile force in the cross direction (CD) 650 N; and permeability coefficient 0.00202 m/s. The implemented type of protection turned out to be flexible, durable, and hydraulically efficient. The protection of the ditch was combined with a stone drainage system (stone with a diameter of 10–60 mm) 3 m wide and 0.2 m thick in a geotextile cover, which was located at the base of the air slope. Protection of the dam ditch and stone drainage with geotextile at the Białobrzegi dam are presented in Figure 20. Applications include geotextiles in the renovation works of the dam contributed to the creation of security, which means that the dam stands safely to this day.

Model tests on the use of geotextiles have been conducted by Lee et al. [114], among others. In their study, a conduit crack was modeled at the risk of internal erosion. This case was analyzed using a large-scale model test and three-dimensional seepage deformation analysis. Based on the analyses conducted, a model was proposed that can reduce internal erosion by applying a layer of sand and geotextile to the upper part of the conduit located near the slope. The analysis results suggest that internal erosion can be stopped if the water pressure acting intensively on the pipe break is dispersed by the drainage layer. However, these are only model studies not supported by experience, although the results seem reliable.

### 3.2. Internal Erosion in Drainage Systems of Buildings and Areas

Internal erosion is a phenomenon that occurs not only in embankment dams but also in drainage systems that drain buildings and areas. This phenomenon is often ignored in this type of system. This is probably due to the smaller consequences that internal erosion causes in the subsoil of these systems.

Internal erosion in drainage systems of buildings and areas is a phenomenon that leads to the inefficiency of these systems and, consequently to the damage of the protected construction and areas. Construction drainage involves building an installation whose task is to lower the water level and pressure by taking it over and draining water from the protected area to the receiver. Cases in which the installation concerns the drainage of buildings, structures, industrial and recreational areas fall into the category of permanent drainage. The implementation of such solutions should be effective enough to ensure, in addition to lowering the groundwater level, the stability of protected building structures. The protection of a single building or a group of buildings against the inflow of groundwater is most often provided by perimeter drains in a horizontal arrangement. Proper selection of the drainage method and the correct use of geotextiles guarantee the effectiveness of the adopted engineering solutions.

Flooding of buildings is mainly related to high groundwater levels in permeable soils and the presence of layers of poorly permeable formations in the subsoil [115]. In particular, their shallow location affects the occurrence of groundwater and near-surface water. The level of these waters and their dynamics depend largely on precipitation and the prevailing climate. Therefore, after heavy rainfall, these waters may cause periodic flooding, which will disappear due to field evaporation. Despite deep impermeable layers and a low groundwater level, local flooding of basements may occur. This may be due to the presence of lenses of much lower permeability in permeable formations in the vadose zone. When seepage water infiltrating deep from the subsoil surface encounters such a lens, it stops at its ceiling and is stored in the pores above it [116]. Such accumulation of free water within the aeration zone is called suspended water. Sometimes there is a problem that foundations and walls get wet and the area becomes marshy with capillary water, even though the free groundwater level is at a safe depth. This situation can be observed when there are fine-grained soils at the foundation level, in which the pores form very narrow cracks and channels. The water then rises due to the action of intermolecular forces, creating a capillary rise zone is created between the saturation and aeration zones [117,118]. The height of the capillary can reach several meters, depending on the soil grain size, mineral and organic content, water chemistry and temperature. There are also cases where deep water with a low water table can cause flooding. The groundwater level rises and forms a common level with the deep water only when there is a hydraulic connection between these two types of water. Aquifers interact with each other through hydrogeological windows and aquifer outcrops. Flooding can also occur when there is a layer of well-permeable soil with a high water table below the poorly permeable surface layer. The pressure of the water in the pores will then cause it to infiltrate the pores of the overlying poorly permeable soil, causing it to become excessively wet [119,120].

Perimeter drainage is a system of drains that surrounds the facility to be drained and limits the flow of water to the foundations. It is usually a closed band that protects both the vertical surfaces of the foundation walls and the horizontal surfaces of the basement and footings. It is the most frequently used system for drainage of building sites, buildings complexes or individual buildings. The main element of the drainage system is the overall circumference of the pipes’ drainage holes (so-called drainpipes). This solution allows to collect of water flowing from all directions, i.e., both rainwater seeping through the ground and groundwater, which periodically raises its level.

Drainage pipes at the highest drainage points and the junctions of two or more pipes are connected to sewage wells or special drainage wells. In the lower part of the drainage well, there is usually a sedimentation tank that protects the drainage system from clogging. The water from the perimeter drainage should be removed beyond the boundaries of the plot in such a way as to minimize the risk of its repeated inflow (e.g., by discharging it into a storm sewer or a watercourse).

**Figure 21:**
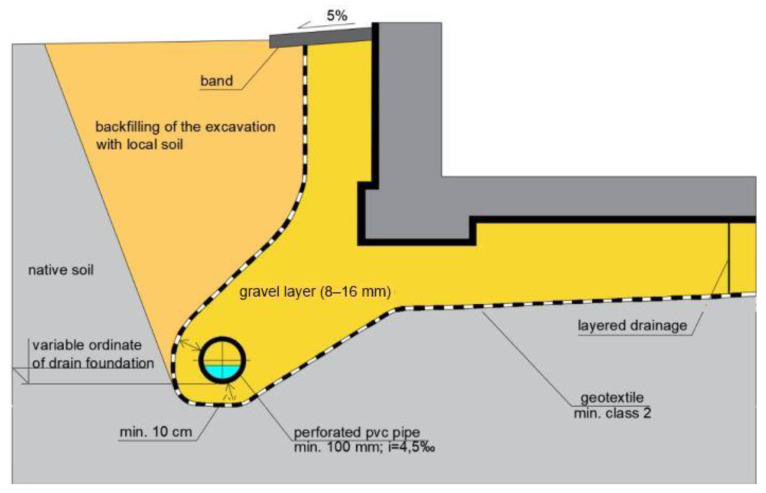
Location of geotextile in perimeter drainages in a newly constructed building.

**Figure 22:**
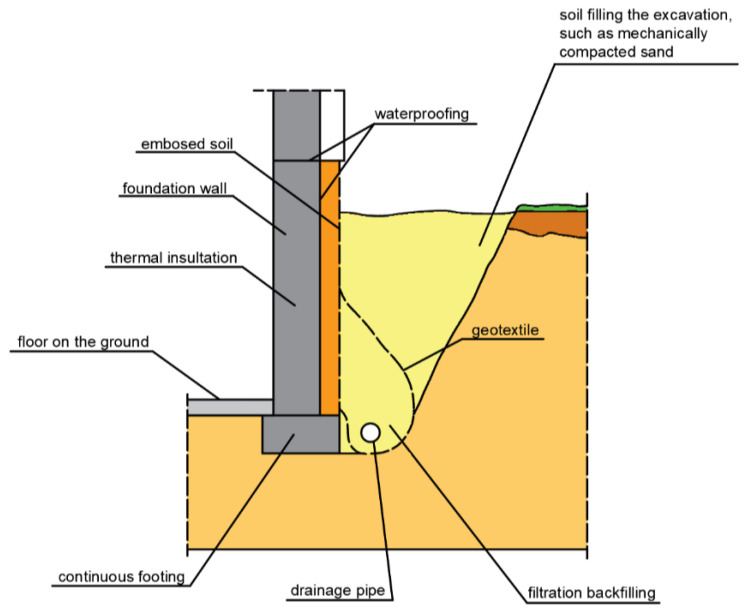
Location of geotextile in perimeter drainages in an existing building.

In perimeter drains, water flowing toward the drains displaces small soil particles due to flow pressure. This causes internal erosion. They are transported through soil pores and perforations into the pipelines. The washing out of particles by the flowing water (suffocation) can lead to the formation of cavities, excessive settlement of the subsoil and siltation of the pipelines. To prevent internal erosion in the drainage of general construction works, granular materials of mineral origin are used: sand, gravel, and sand after sifting out fine fractions, as well as geotextile filtration layers. Geotextiles are most often used to separate the soil layers of the native subsoil from the filtration layer, thus preventing internal erosion, to which fine-grained and interlayered soils are particularly vulnerable. The geotextile is a suitable adaptation material in such cases because it is permeable to water, but at the same time provides a tight barrier to sand and silt. The role of the geotextile is to protect the drainage from siltation and plant root penetration. In existing buildings where drainage is required, the geotextile is placed over the backfill and folded over the exterior wall insulation (Figure 21). In cases where there is possibility of siltation of the drainage from below, the geotextile should be placed directly on the bottom of the excavation, on the pre-prepared layer of sand (Figure 22). The situation shown in Figure 22 is possible in cases where the drainage is carried out simultaneously with the construction of the building.

In this type of drainage system, geotextile is also used to cover perforated pipes. Coconut fiber can also be used instead of geotextile, but this is a rarely used solution. If the pipes are installed in a lagging, there is no need to use a gravel backfill and geotextile to separate the native subsoil from the drainage system (although its additional use will improve the rate of water collection through the pipe).

Failure to separate the native subsoil from the filtration backfill with a layer of geotextile soil or to protect the drainage pipes with a geotextile cover may result in the occurrence of internal erosion of the subsoil, which will reduce the effectiveness of the drainage or, in the worst case, interrupt its operation, thus damaging the protected facilities.

French drains, also known as trench drains, are used to drain water from the subsoil by means of a trench filled with aggregate and isolated from the surrounding soil with geotextile. In addition, a drainpipe is often used to accelerate the flow of water. They can be used to drain water around a building, from pavement layers, and roadsides or terrain surfaces. This technique is used to protect foundations from moisture, prevent water from stagnating in the subsoil, and protect the area from internal erosion.

**Figure 23:**
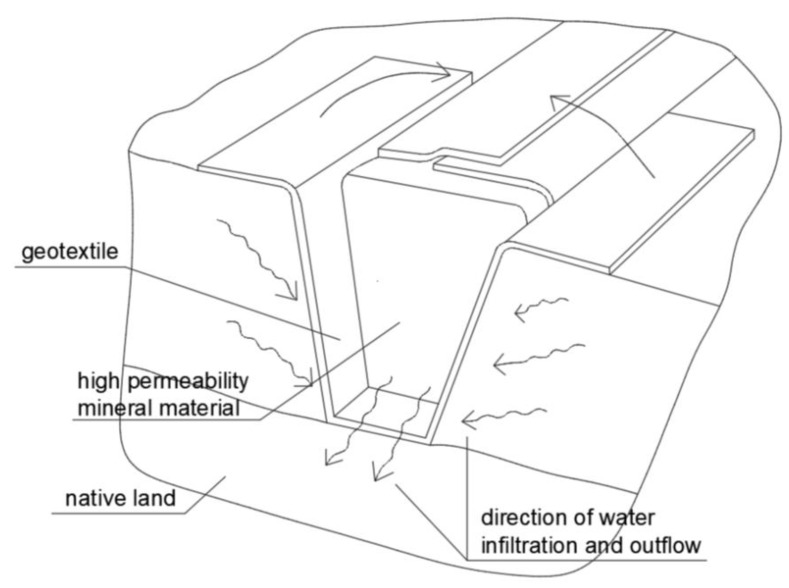
Location of geotextile in the French drain system.

The French drain consists of a filter made of mineral material: aggregated crushed stone surrounded by geotextile that prevents small soil particles from entering the filter. The type of geotextile should be selected according to the conditions, location, and design requirements. Needle-punched nonwoven geotextile is the best choice for reliable, long-lasting operation. A cross section through the typical French drain system is shown in Figure 23.

Correct operation of the drainage is ensured by the proper selection of geotextile and its correct arrangement. The basis of this type of drainage is to surround the aggregate with geotextile and to place a pipe that accelerates the flow of water inside the aggregate.

A French drain ensures good water transport to the receiver, significantly reduces construction and operating costs, and extends the period of failure-free operation, mainly due to the elimination of soil siltation, in comparison with drainage carried out by traditional technology, i.e., circular drainage. The operating principle of the French drain is to reduce the speed of water by allowing it to flow through a very large number of pores on the surface of the geotextile. This reduces the energy of the infiltrating water, which in turn prevents soil particles from entering the drain, thus preventing internal erosion.

French drains can also act as a barrier to pollutants by preventing them from seeping into the ground, which is especially important in industrial areas.

Chambers, tunnels, and infiltration boxes are used to distribute rainwater collected from paved areas to the subsoil. This solution is also used in the design of rainwater management systems for homes and gardens to prevent excessive accumulation of rainwater in rainwater tanks. The system safely captures overflowing rainwater and then absorbs and evenly distributes it to the subsoil. These are systems where internal erosion can also occur.

Infiltration chambers, tunnels, and boxes allow water to be retained in the subsoil instead of being discharged into the recipient, which is why they are becoming increasingly popular, especially in cities and in regions and properties without access to a sewerage system. A prerequisite for the use of these solutions is a well-permeable subsoil and a low groundwater level. Infiltration chambers are characterized by high durability; therefore, they are most often placed under roads and other surfaces that may be subject to higher loads. Infiltration tunnels are less durable than chambers and are used under lawns, sidewalks, and garage driveways. Infiltration boxes are the least durable, have the lowest load-bearing capacity, and are used under lawns.

The condition for the proper operation of this type of system is the use of aggregate and geotextile in the place of water infiltration. The geotextile makes it possible to separate the subsoil from the seepage system, thus protecting it from being washed out and preventing soil particles from entering the chamber, tunnel, or box. Appropriate distribution of the geotextile prevents soil particles from being washed out under the water distribution system, and thus from causing internal erosion in the subsoil. In this way, the use of geotextile ensures the proper and long-lasting operation of the water distribution system.

## 4. Discussion

Given the multiplicity of erosion types that constitute the subject of research and practical engineering problems, universal solutions are particularly desirable. This is facilitated by the wide range of applications of different materials and the possibility of putting them together in modules. Thanks to them, it is possible to achieve better technical results and significant financial savings through the project optimization procedure and to minimize the negative impact of the stage on the environment.

When analyzing the environmental aspect, one often looks at the material from which geosynthetics are manufactured. It is generally assumed that the material from which geosynthetics are made is synthetic polymer material, which could pollute our environment and cause ecological problems. A literature review [121] concluded that there is a lack of a clear study on the environmental impact of geosynthetics. Although numerous studies are available on the potential applications of geosynthetics in landscape engineering, the literature review performed by the authors supports this claim. Besides the target function during use, the production of geosynthetic materials itself is a process, affecting the surrounding land. The manufacture of these materials involves the emission of carbon dioxide and other pollutants at the extraction stage and nitrogen oxides, sulfur oxides, and volatile organic compounds [122]. Moreover, production processes require a considerable amount of water, both for cooling equipment and for the production process itself [123].

Geosynthetic production also generates waste in the form of solids and wastewater [122,124]. If waste is not properly managed at both the production and disposal stages, it can be sent to landfills, where it will linger for many years, contributing to soil and groundwater contamination [125].

Geosynthetics have a long lifespan. The process of their decomposition is far longer than maintaining the optimal properties of a given material. At the production and disposal (or recycling process) stage, the importance of conducting ongoing maintenance inspections and monitoring water contamination [126], including the content of salts and other mineral substances in leachates, should be emphasized.

One should also look at the tasks geosynthetics fulfill in the environment after their use. The correct use of geosynthetics contributes greatly to environmental protection, by solving many environmental problems, such as erosion. If they are properly manufactured and disposed of, they support sustainable development and must be taken into account in future investments in all market sectors.

Moreover, for many years, researchers worldwide have emphasized the effectiveness of natural solutions in civil and hydraulic engineering. Geosynthetic products used for erosion protection are not only synthetic polymers made of plastics but also materials made of natural polymers that are biodegradable. These include, for example, biotextiles made of natural fibers with grass seeds placed in the ground cover, fabrics and nets made of natural fibers, and mats made of natural polymers. These materials are most often used to protect road slopes and provide protection against erosion in the short term, up to two years. After decomposition, they provide an additional dose of fertilizer for the vegetation growing on the slopes and thus contribute to improving the quality of the natural environment.

Notably, it is important to mention that natural solutions are frequently available in the vicinity of implemented investments, resulting in lower costs and an efficient and less intrusive construction process organization. In this regard, the application of, e.g., greenery placed in the top layer of geocells is also an opportunity to create habitats friendly to living organisms and blue–green infrastructure space and constitutes an important step towards sustainable landscape engineering. However, the list of plant species is not developed in its final form. It should be noted that such solutions can be a tool to compensate for the negative environmental impact of the investment by, among other things, improving the local water balance and soil quality. Therefore, combining natural solutions with geosynthetics should be the focus.

## 5. Conclusions

Water erosion is one of the most difficult processes to control in hydrotechnical engineering structures. It occurs on the surface of slopes, inside buildings, and in the ground. The effect of uncontrolled erosion is destructive processes, leading to degradation, damage, and as a result, sometimes to the destruction of the entire building. The phenomenon is described theoretically and experimental research is carried out in field and laboratory conditions. The selection of erosion protection elements and materials for their production requires the use of appropriate selection criteria, which are proven for mineral filters. In the case of using artificial materials (geosynthetics) for anti-erosion protection, the selection criteria are still not clear, but the use of these materials has many possibilities and benefits.

Geosynthetic elements allow erosion control measures to be placed even in difficult hydraulic conditions where other methods may fail. Many external erosion control structures would be impossible to construct without geosynthetic support.

Synthetic materials do not adversely affect the chemical or ecological status of the watercourse, and often even have a positive effect on its ecosystem, but they do have one significant disadvantage. In most cases, immediately after the completion of the work, the constructed structure does not yet perform its intended function, and the permissible stresses it can bear are small. Significant strength is sometimes achieved only after a year. This is usually due to the need for rooting and plant growth.

The choice of a suitable solution depends on many factors, such as the slope, the hydrological conditions, or the type of soil in which the embankment is built, but above all on the nature and intensity of the impact of the material on the naturalness and condition of the watercourse.

Geosynthetics also play an important role in preventing internal erosion. Geosynthetics are now commonly used in the construction of embankments or drainage systems. These are materials that perform many functions in structures, but it is so important to determine which primary function the geosynthetic will perform in the construction. The soil and hydraulic parameters of the geosynthetics, such as characteristic opening size and water permeability normal to the plane, must be taken into account when using geosynthetics in drainage systems. This is the only way to ensure proper design and long-term performance of the structure.

Future research should focus primarily on establishing clear criteria for selecting anti-erosion geosynthetics. It is important to remember that only the correct selection of geosynthetics in an engineered structure will ensure its long-term performance. As new geosynthetic products are introduced, it is necessary to use modern laboratory techniques to determine their suitability for engineered structures. Certainly, one of the directions for further research is to investigate the possibility of using geosystems in construction. By combining the properties of several products, they can effectively influence, for example, the improvement of slope stability of embankments or the prevention of erosion.

Further suggestions for future research include understanding the overall erosion system and the use of polymers to prevent this process. To this end, detailed studies should be conducted to assess the likelihood of erosion occurring in existing systems, which should be determined in quantitative and qualitative terms. The use of analytical methods, including the development of uncertainty quantification, should be sought. It is also important to improve decision-making criteria based on retrospective analysis of erosion events when good-quality data are available, as well as development of surveillance and monitoring techniques that are designed to identify internal erosion failures. It is also very important to use numerical methods to model all phases of the process leading to breaching and validating them in real case studies.

When analyzing the impact of geosynthetics on the environment, attention should be paid to the benefits that flow to the environment from their use, not to the material from which they are produced. The production of geosynthetics using modern technologies does not negatively affect the environment, and their correct use in the environment supports sustainable development.

## Figures and Tables

**Table 1:** Polymers used in the manufacture of geosynthetics.

Polymer	Repeating Unit	Density (g/cm^3^)
Polypropylene (PP)	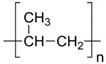	0.90–0.92
Polyester (PET)	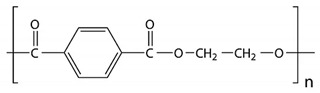	1.22–1.38
Polyethylene (PE)	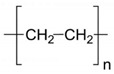	0.91–0.97
Polyamide (PA) (nylon 6/6)	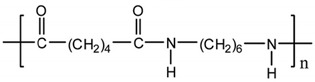	1.14
Polyvinyl chloride (PVC)	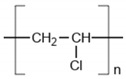	1.40
Polystyrene (PS)	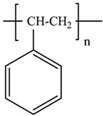	0.96–1.05

**Table 2:** Geosynthetics and the polymers used in their production [24].

Geosynthetics	Polymer
geotextiles, geomembranes, geogrids, geocomposites	Polypropylene (PP)
geotextiles, geogrids	Polyester (PET)
geotextiles, geomembranes, geogrids, geonets, geocomposites	Polyethylene (PE)
geotextiles, geocomposites, geogrids	Polyamide (PA) (nylon 6/6)
geomembranes, geocomposites	Polyvinyl chloride (PVC)
geocomposites	Polystyrene (PS)

**Table 3:** Geosynthetic parameters and the commonly used methods for their determination.

Parameters	Method
Water permeability normal to the plane (m/s)	The constant head method or the falling head method
Characteristic opening size (μm)	Wet sieving test method
Thickness under 2 kPa (mm)	Measuring the distance between a reference plate on which the specimen rests and the contacting face of a parallel pressure foot applying a given pressure to the specimen
Mass per unit area (g/m^2^)	Weighing test specimens of known dimensions
Fiber diameter (μm)	Scanning electron microscope
Tensile strength—MD (kN/m)	Wide-width tensile test
Tensile strength—CMD (kN/m)	Wide-width tensile test
Elongation at maximum load—MD (%)	Wide-width tensile test
Elongation at maximum load—CMD (%)	Wide-width tensile test

## Data Availability

Not applicable.
